# The Effect of Temperature and Perfusion Time on Response, Toxicity, and Survival in Patients with In-transit Melanoma Metastases Treated with Isolated Limb Perfusion

**DOI:** 10.1245/s10434-018-6459-9

**Published:** 2018-05-15

**Authors:** Dimitrios Katsarelias, Erik Rådbo, Ilan Ben-Shabat, Jan Mattsson, Roger Olofsson Bagge

**Affiliations:** Department of Surgery, Institute of Clinical Sciences, Sahlgrenska Academy at the University of Gothenburg, Sahlgrenska University Hospital, Gothenburg, Sweden

## Abstract

**Background:**

Isolated limb perfusion (ILP) is used to treat in-transit metastases of melanoma of the extremities when surgical excision is not possible. The optimal setting concerning temperature and perfusion time is unknown. The purpose of this study was to analyze these factors concerning their effects on response, toxicity, and survival.

**Methods:**

A retrospective analysis of 284 consecutive stage III melanoma patients treated with melphalan ILP for the first time in our institution, during a 31-year period (July 1986–May 2017), was performed. Our series was divided in four time periods, according to perfusion temperature and duration. Demographical data, stage, number, and size of lesions were retrieved from our prospective database.

**Results:**

Overall response (OR) rate 83% and a complete response (CR) rate of 59%. Significant predictive factors for CR in multivariate analysis were non-bulky tumor, fewer metastases, and a perfusion time of 120 min. Predictive factors for increased local toxicity were femoral ILP and higher perfusion temperatures. The median overall survival was 30 months, and the independent negative prognostic factors were lymph-node status, bulky tumors, response, upper limb perfusion, and 120 min perfusion at 39–40 °C.

**Conclusions:**

Modern ILP uses diminished perfusion time and lower temperature, leading to a decrease in toxicity. However, our data also show a decrease in response, which indicates that optimal perfusion time and temperature regimen remain to be determined.

## Background

Approximately 5–10% of patients with recurrence of malignant melanoma develop lymphatic dissemination manifested as in-transit metastasis.^1^ The initial treatment option is surgical excision; however, in case of numerous lesions or short intervals between the appearances of new lesions, alternative treatment modalities should be considered.

The technique of isolated limb perfusion (ILP) was pioneered in the 1950s by Creech and Krementz.^2^ Compared with systemic administration, ILP achieves very high tissue concentrations of the alkylating agent melphalan. An early pharmacokinetic study using melphalan in an ILP setting showed that high peak perfusate concentrations were achieved (6–115 mg/ml) and that these levels could be 20–100 times higher than the peak levels achieved with the usual intravenous doses of melphalan.^3^ In 1967, Cavaliere reported the effects of ILP using only hyperthermia in 22 patients with recurrent extremity tumors. The duration of hyperthermia (> 40 °C) ranged from 50 min to almost 7 h. Twelve of the patients were alive without evidence of disease at 3–28 months of follow-up.^4^

In 1969, Stehlin increased perfusion time from 45 to 120 min and heated the perfusate to 46.1 °C. Together with externally applied heat and wrapping, they reached skin temperatures of 42.2 °C. These changes resulted in higher tumor response but also increased complication rates.^5^ The method has since then been refined and current results for ILP with melphalan (M-ILP) show overall response rates (ORR) ranging between 65 and 100%, with a complete response (CR) rate between 25 and 76%.^6^

In Sweden, ILP treatment was initiated in the 1970s. Initially, the perfusion time was 120 min with a tissue temperature of 41–41.5 °C (true hyperthermia), which in 2002 was lowered to 39–40 °C. The perfusion time was lowered to 90 min in 2006 and then finally to 60 min in 2012 keeping perfusions temperature between 39 and 40 °C (mild hyperthermia). The administration of melphalan was also changed, from three bolus doses, in the earlier periods, to 20 min infusion in 2012, and this is still in use in our practice.

The purpose of this study was to analyze retrospectively the effect of temperature lowering and diminishing of perfusion time on response, toxicity, and survival for melanoma patients with in-transit metastases treated with melphalan-only ILP in our institution.

## Patients and Methods

### Patients

Over a 31-year period (July 1986–May 2017), a total of 284 consecutive patients with in-transit metastases of malignant melanoma (stage III), were treated with ILP for the first time in our institution. In the beginning of this study period, TNF-alpha was not available, and patients who later received TNF-alpha due to bulky melanoma were excluded not to induce a further bias between the treatment groups. There were 166 females and 118 males with a median age of 70 years (range 23–95). A total of 171 patients (60.2 %) had in-transit metastases only (N2c), and 113 patients (39.8 %) had lymph node metastases (N3) before or at the time of ILP. Bulky melanoma (lesions > 3 cm) was present in 36 patients (12.7%), and 88 patients (31.0%) had 10 or more lesions (Table 1). No patients received any adjuvant systemic therapies.

**Table 1:** Patient and tumor characteristics

Sex	
Female	166 (58.5%)
Male	118 (41.5%)
Age. median (range), years	70.5 (23–95)
N-stage (%)	
N2c	171 (60.2%)
N3	113 (39.8%)
Vessel (%)	
Upper extremity	34 (12.0%)
Femoral	151 (53.2%)
External iliac	99 (34.9%)
Perfusion time/temp (%)	
60 min/ 39–40 °C	91 (32.0%)
90 min/ 39–40 °C	86 (30.3%)
120 min/ 39–40 °C	17 (6.0%)
120 min/ 41–41.5 °C	90 (31.7%)
Number of metastases (%)	
1	39 (13.7%)
2–3	69 (24.3%)
4–10	81 (28.5%)
> 10	88 (31.0%)
Missing	7 (2.5%)
Largest metastasis (%)	
Nodular (< 3 cm)	237 (83.5%)
Bulky (> 3 cm)	36 (12.7%)
Missing	11 (3.9%)

Baseline data, as well as data concerning response and toxicity, were retrieved from a prospectively kept database, further completed with data from patient medical records. Data concerning survival was retrieved from the Swedish National Cause of Death Register. The study was approved by the Regional Ethical Review Board at the University of Gothenburg (Dnr 721-08).

### ILP Technique

The patients underwent ILP via an axillary, brachial, or subclavian vascular approach for upper extremity (*n* = 34) and via the external iliac (*n* = 99) or femoral (*n* = 151) approach for the lower extremity (Table 1). Limb isolation was achieved through clamping and cannulation of the major artery and vein for the extremity under treatment. The cannulas were connected to an oxygenated extracorporeal circuit. From October 2000, continuous leakage monitoring was performed using a precordial scintillation probe (Medic View, Sweden) to detect and measure leakage of technetium-99m labelled human serum albumin (Vasculosis, Cis-Bio International, Gif-sur-Yvette, France), which was injected into the perfusion circuit. The dose of melphalan was calculated as 13 mg/L perfused tissues for upper limb and 10 mg/L perfused tissues for lower limb.

### Time Periods

Between 1986 and 2002, the perfusion time and the highest tissue target temperature was 120 min and 41–41.5 °C respectively. In 2002, this was changed to 120 min at 39–40 °C, and this temperature was then used onward. In 2006, the total perfusion time was decreased to 90 min, and in 2012, the perfusion time was further decreased to 60 min. Before 2012, the melphalan was given as three bolus doses, with 50% of the total dose administered initially and the remaining 50% administered in two equivalent doses at 30-min intervals (total 60 min). In 2012, the administration of melphalan was changed into a 20-min infusion in the perfusate, followed by 40-min perfusion.

### Response

Clinical responses were evaluated and reported as the response at 3 months using the WHO criteria.^7^ For a response to be considered as a complete response (CR), all lesions should have been clinically not detectable at the time of clinical examination. Partial response (PR) was defined as a clinical decrease of more than 50% of the total tumor burden both in terms of number of lesions or diameter. Progressive disease (PD) was defined as an increase of more than 25% in existing lesions or the appearance of new lesions not previously present. Stable disease (SD) was defined as a result where none of the abovementioned criteria for CR, PR, or PD were met.

### Local Toxicity

Local toxicity was measured according to Wieberdink and classified from I to V, where I is no reaction, II slight erythema and/or edema, III is considerable erythema and/or edema with some blistering, IV is extensive epidermolysis and/or obvious damage to deep tissues, and V induces a reaction that may necessitate amputation.^8^

### Statistical Evaluation

Overall survival (OS) was defined as the time from ILP to death or last follow-up. Survival estimates were made according to the Kaplan-Meier method and prognostic factors for OS were analyzed using Cox regression. Predictive factors for response and toxicity were analyzed using logistic regression. A *p* value < 0.05 was considered statistically significant. All data were analyzed by using SPSS version 22 (SPSS, Chicago, IL).

## Results

### Response

Clinical response was evaluable in 268 patients (94.4%). Two patients were not included due to early death (earlier than 3 months), and 14 patients were excluded due to lack of reliable response records. In total, 223 patients (83.2%) had an ORR, of which 167 patients (58.8%) had a CR. Significant predictive factors for CR in univariate analysis were a total number of metastases less than ten and a longer perfusion time (120 min) under mild hyperthermia (39–40 °C). In the multivariate analysis, both the number of metastases, as well as longer perfusion time (120 min) at 39–40 °C, were proven statistically significant (Table 2). For overall response, the only significant predictive factor was gender; males had a lower response (odds ratio = 0.43; *p* = 0.02).

**Table 2:** Univariate and multivariate logistic regression of clinical predictive factors for complete response after isolated limb perfusion

Variables	Univariate analysis			Multivariate analysis		
	OR	95% CI	*p* value	OR	95% CI	*p* value
Age (year)	1.01	0.99–1.03	0.24	1.02	1.00–1.04	0.09
Gender (female vs. male)	0.86	0.58–1.58	0.86	0.82	0.46–1.47	0.51
N-stage (N2c vs. N3)	0.80	0.48–1.32	0.37	0.94	0.53–1.67	0.83
Size (nonbulky vs. bulky)	0.83	0.40–1.73	0.62	0.57	0.24–1.35	0.20
Number of lesions						
1	1			1		
2–3	0.56	0.20–1.59	0.28	0.54	0.18–1.63	0.28
4–10	0.38	0.14–1.03	0.06	0.32	0.11–0.93	0.04
> 10	0.13	0.05–0.33	< 0.0001	0.10	0.03–0.30	< 0.0001
Vessel						
Femoral	1			1		
Upper extremity	1.44	0.62–3.37	0.40	0.89	0.35–2.30	0.81
External iliac	0.88	0.52–1.50	0.63	0.74	0.35–1.55	0.42
Perfusion time/temp						
60 min /39–40 °C	1			1		
90 min /39–40 °C	1.75	0.93–3.29	0.08	1.84	0.91–3.61	0.09
120 min /39–40 °C	4.16	1.12–15.5	0.03	4.85	1.19–19.8	0.03
120 min /41–41.5 °C	1.69	0.92–3.12	0.09	1.50	0.64–3.48	0.35

### Toxicity

Data on local toxicity was available in 270 patients (95%). Reliable data were missing for 14 patients, and they were excluded from the analysis. The distribution between Wieberdink grades through the entire 31-year period were: grade I 4.4%, grade II 62.6%, grade III 24.4%, grade IV 8.1%, and grade V 0.4%. In multivariate analysis comparing Wieberdink I–III versus IV–V, perfusion at 41–41.5 °C for 120 min (1986–2002) had a higher rate of severe toxicity (grade III–V) with an odds ratio of 3.9 (*p* = 0.04), whereas external iliac perfusions had a significantly lower rate (OR 0.25; *p* = 0.03; Table 3). Similar results were obtained in the multivariate analysis comparing Wieberdink I–II versus III, where higher temperature (41.5 °C at 120 min) had more grade III toxicity (OR 2.59, *p* = 0.05), whereas both external iliac and brachial perfusions had lower rates of grade III toxicity (brachial OR 0.22 *p* = 0.01; external iliac OR 0.23, *p* = 0.001) compared with femoral perfusions.

**Table 3:** Univariate and multivariate logistic regression of clinical predictive factors for local toxicity (Wieberdink I–III vs. IV–V) after isolated limb perfusion

Variables	Univariate analysis			Multivariate analysis		
	OR	95% CI	*p* value	OR	95% CI	*p* value
Age (year)	1.00	0.97–1.03	0.83	0.98	0.95–1.02	0.29
Gender (female vs. male)	0.76	0.31–1.86	0.54	0.95	0.36–2.52	0.92
N-stage (N2c vs. N3)	0.53	0.20–1.39	0.19	0.53	0.19–1.47	0.22
Size (nonbulky vs. bulky)	1.09	0.30–3.88	0.90	0.96	0.23–4.06	0.96
Number of lesions						
1	1			1		
2–3	0.39	0.10–1.55	0.18	0.45	0.10–2.01	0.30
4–10	0.44	0.12–1.62	0.22	0.48	0.11–2.05	0.32
> 10	0.64	0.20–2.12	0.47	0.85	0.21–3.49	0.82
Vessel						
Femoral	1			1		
Upper extremity	0.56	0.12– 2.60	0.46	0.51	0.10–2.73	0.43
External iliac	0.56	0.21–1.51	0.26	0.25	0.07–0.87	0.03
Perfusion time/temp						
60 min /39–40 °C	1			1		
90 min /39–40 °C	0.47	0.12–1.88	0.29	0.61	0.15–2.54	0.50
120 min /39–40 °C	0.72	0.08–6.29	0.77	0.94	0.10–8.78	0.95
120 min /41–41.5 °C	1.83	0.68–4.88	0.23	3.90	1.08–14.1	0.04

### Survival

**Fig. 1:**
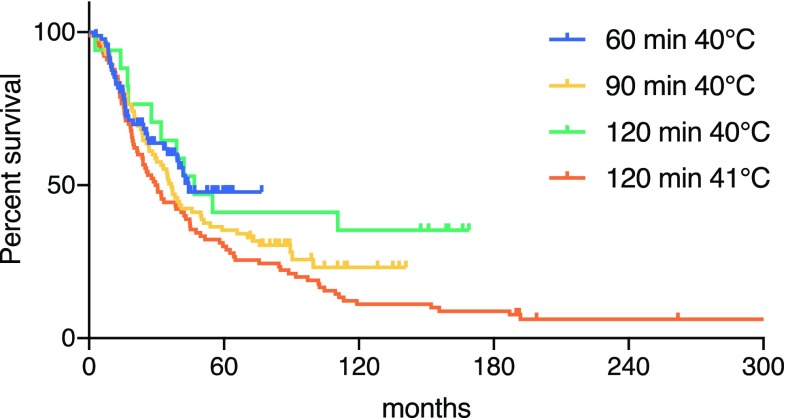
Overall survival after isolated limb perfusion

Survival data were available for all 284 patients included in the study. The 2, 5, and 10-year overall survival (OS) was 64, 36, and 19% respectively, with a median OS of 38 months. The median OS was 44 months in the 60-min ILP at 39–40 °C group (2012–2017), 36 months for the 90-min ILP at 39–40 °C group (2006–2012), 47 months for the 120-min ILP at 39–40 °C group (2002–2006), and 30 months for the 120-min ILP at 41–41.5 °C group (1986–2002; *p* = 0.06; Fig. 1). In multivariate analysis, significant negative prognostic factors were increasing age, positive lymph-node status, bulky disease, brachial ILP, and response after ILP and ILP for 120 min at 41–41.5 °C (Table 4). When excluding response and toxicity from the survival analysis, the significant prognostic factors were age (HR 1.02, *p* = 0.047), positive lymph-node status (HR 1.52, *p* = 0.01), presence of bulky disease (HR 2.06, *p* = 0.002), more than ten metastases (HR 1.85, *p* = 0.02), and ILP for 120 min at 41–41.5 °C (HR 1.84, *p* = 0.02).

**Table 4:** Univariate and multivariate Cox analysis of clinical predictive factors for overall survival after isolated limb perfusion

Variables	Univariate analysis			Multivariate analysis		
	OR	95% CI	*p* value	OR	95% CI	*p* value
Age (years)	1.02	1.01–1.03	0.007	1.02	1.00–1.03	0.03
Gender (female vs. male)	1.24	0.93–1.66	0.14	1.12	0.79–1.71	0.52
N-stage (N2c vs. N3)	1.50	1.13–2.00	0.005	1.99	1.41–2.61	< 0.0001
Size (Non-bulky vs. bulky)	1.80	1.20–2.70	0.005	2.56	1.59–4.10	0.0001
Number of lesions						
1	1			1		
2–3	0.93	0.57–1.51	0.76	1.24	0.71–2.03	0.45
4–10	1.13	0.71–1.79	0.61	1.44	0.85–2.23	0.18
> 10	1.43	0.90–2.28	0.13	1.55	0.87–2.59	0.12
Vessel						
Femoral	1			1		
Upper extremity	1.18	0.74–1.88	0.49	1.78	1.01–2.89	0.046
External iliac	1.30	0.96–1.75	0.09	1.24	0.83–1.82	0.29
Perfusion time / temp						
60 min /39–40 °C	1			1		
90 min /39–40 °C	1.24	0.82–1.87	0.32	1.24	0.73–2.11	0.43
120 min /39–40 °C	0.79	0.40–1.57	0.51	1.24	0.57–2.51	0.59
120 min /41–41.5 °C	1.55	1.04–2.32	0.03	2.03	1.16–3.36	0.01
Response						
CR	1			1		
PR	1.52	1.05–2.21	0.03	1.69	1.12–2.81	0.01
SD	1.73	1.08–2.77	0.02	1.51	0.88–2.72	0.13
PD	3.04	1.66–5.57	< 0.001	4.39	2.04–9.66	0.0001
Local toxicity						
Wieberdink I	1			1		
Wieberdink II	1.06	0.49–2.29	0.88	0.96	0.38–2.38	0.92
Wieberdink III	1.20	0.54–2.67	0.65	1.26	0.48–3.29	0.64
Wieberdink IV	0.82	0.33–2.05	0.68	0.80	0.27–2.34	0.68

## Discussion

The purpose of this study was to perform a retrospective analysis on the effect of temperature and perfusion time on response, toxicity, and survival after first-time ILP with melphalan only for melanoma in-transit metastases. The major limitation of this study was its retrospective design. However, it comprises a single-institution experience with few involved surgeons and standardized technique during the period under examination. To answer the research question, the consecutive material at our center was divided into four distinct time periods, with changes in perfusion time, perfusion temperature, and way of melphalan administration (bolus doses vs. 20 min infusion).

Concerning response, there was an increased response rate associated to longer perfusion time (120 min). When comparing 60 min perfusion at 39–40 °C (mild hyperthermia) to the 90 min perfusion, there was a trend towards better response in the longer perfusion group with an odds ratio of 1.84 (*p* = 0.09), and this became significant when 60-min perfusion was compared with 120-min perfusion under mild hyperthermia with an odds ratio of 4.85 (*p* = 0.03). Interestingly, a 120-min perfusion at 41–41.5 °C (true hyperthermia) did not achieve a higher response rate. Why higher temperature perfusion did not result in the same or higher response rate can only be speculated upon, but it might have been due to factors that changed through the years that were not accounted for in this analysis. Alternatively, we have previously shown that immunological factors are important for response after ILP, and it might be that true hyperthermia is not as effective to activate the immune system.^9^–^11^ We currently use a 60-min perfusion protocol, but data from this study point towards a better response rate using mild hyperthermia for a longer perfusion duration. The effect could be partly due to the changes from a divided bolus administration to a continuous infusion for 20 min.

Following ILP, local toxicity often is evident. Common signs of local toxicity are discomfort, erythema, and edema, which occurs in most of the patients. In some, more severe cases, other side effects of the treatment can occur, such as temporary loss of nails and hair, blistering, impermanent neuralgia, rhabdomyolysis, and compartment syndrome.^12^ Several factors have been shown to be associated with local toxicity.

It has already been demonstrated that perfusion temperatures more than 40 °C increase toxicity, a finding that also could be confirmed in this series for patients treated with a 120-min perfusion at 41–41.5 °C.^13^–^16^ It has been demonstrated that hyperthermia mediates an increased uptake of chemotherapeutics through changes in tumour blood flow and cellular permeability.^17^ During ILP, an increase in temperature from 37 to 39.5 °C doubles the concentration of cisplatin in tumours while at the same time decreasing the concentration in surrounding healthy tissue.^18^ Hyperthermia also acts synergistically with melphalan leading to an increased toxicity in human melanoma cell lines.^19^ Previous reports showed that tissue temperatures of 41.5° or more generates a high response rate, but this could not be confirmed in this material.^20^ As a compromise between response rate and toxicity, our current standard is to use tissue temperatures of mild hyperthermia (39–40 °C).

It has previously been shown that a more proximal isolation for both upper and lower extremity gave a higher rate of local toxicity.^15^ However, in our series, iliac ILP was an independent predictive factor for lower toxicity. This might be due to differences in melphalan concentration between the different levels of isolation. One could speculate that iliac ILPs received a lower dose of melphalan, because it has previously been shown that melphalan concentration, both peak and area under the curve (AUC), affects toxicity.^14^

Negative independent prognostic factors for survival were age, the presence of lymph node metastases, bulky tumors, brachial ILPs, 120-min perfusion at 41–41.5 °C, and not achieving a CR. Although not statistically significant, there was an improvement in survival over time in univariate analysis (Fig. 1). However, only the earliest group (120 min at 41–41.5 °C) was significant in multivariate analysis. This phenomenon is probably because patients are receiving more effective systemic therapies in recent years, which has an impact on overall survival. Although only stage III patients were included in the analysis, some of them have received immunotherapy or BRAF, MEK inhibitors in the later stage of their disease. This has probably contributed to a better overall survival in the later periods.

The results from this retrospective study point toward that prolonged perfusion time increases the response rates without an increase in local toxicity, whereas increased temperature increases the toxicity but not response rate. However, considering the retrospective design of this study, these results must be interpreted with caution. At our institution, we have discussed this and decided not to change our current protocol at this time, maintaining a 60-min perfusion under mild hyperthermia with melphalan infusion for 20 min. The optimal temperature and perfusion time will probably not be analyzed in prospective, randomized trials, and it would take many years and multiple centers to verify the results. More important might be to include the current results in future trial designs, where more urgent research questions can be analyzed. Future research will most probably not just try to maximize response rates, which already are superior to other treatment options, but rather to study combinatorial treatments ultimately leading to cure in this patient population. A very interesting development is the synergistic effect of ipilimumab and isolated limb infusion that was shown in a recent publication.^21^ This kind of combined approaches, together with other locoregional interventions, need to be further investigated to obtain better response rates, lower toxicity, and why not better survival.
